# Screening of Natural Bioactive Metabolites and Investigation of Antioxidant, Antimicrobial, Antihyperglycemic, Neuropharmacological, and Cytotoxicity Potentials of* Litsea polyantha* Juss. Ethanolic Root Extract

**DOI:** 10.1155/2017/3701349

**Published:** 2017-10-30

**Authors:** Nripendra Nath Biswas, Amit Kumar Acharzo, Shams Anamika, Shamsunnahar Khushi, Bishwajit Bokshi

**Affiliations:** Pharmacy Discipline, Life Science School, Khulna University, Khulna 9208, Bangladesh

## Abstract

This study was designed to identify some bioactive phytochemicals from ethanolic extract of roots of* Litsea polyantha* and to evaluate some of its pharmacological activities. Phytochemical tests indicated the presence of reducing sugar, combined reducing sugar, tannins, flavonoids, alkaloids, terpenoids, and phenol. In the antioxidant assay using 2-diphenyl-1-picryl-hydrazyl (DPPH) free radical scavenging method, the IC_50_ value was found to be 82.31 *μ*g/mL. Total content of phenolic compounds, flavonoid, and tannin was found to be 152.69 mg GAE/gm, 85.60 mg QE/gm, and 77.22 mg GAE/gm of dry extract, respectively. In disc diffusion antibacterial assay, the extract exhibited highest zone of inhibition up to 12.25 mm against* Escherichia coli* at the concentration of 500 *μ*g/disc. For brine shrimp lethality bioassay, the extract exhibited LC_50_ 56.082 *μ*g/mL. In* in vivo* antihyperglycemic activity test by oral glucose tolerance test using* Swiss Albino* mice at the oral dose of 250 and 500 mg/kg, the extract showed statistically significant antihyperglycemic effect. Finally,* in vivo, *the extract exhibited the dose dependent CNS depressant effects by reducing the locomotors of* Swiss Albino* mice which was confirmed through three different neuropharmacological activity tests such as open field, hole cross, and hole board test.

## 1. Introduction

Since ancient times, life and diseases are associated with each other. Every year human civilization is facing many new diseases which are many times difficult to treat with conventional drugs. So, it is an urge to find a novel alternative which is potent but less toxic. Scientists are continuously devoted to search for new drug from synthetic as well as natural sources such as minerals, plants, and animals [1]. As plants are readily available to us and are routinely used as foods or other purposes, it is believed that bioactive compound derived from plant may cause less toxicity [2]. Thus, natural products especially those derived from plant have been drawing interest as alternative therapies [3]. Approximately 25% prescribed drugs throughout the world are derived from diverse plant sources [4]. However, the investigation of plants as a source of therapeutic lead compound is still impoverished and there are many new windows for identifying the desired potent drug molecules [3].* Litsea monopetala* Roxb. is the botanical synonym of* Litsea polyantha* Juss. (Lauraceae). It is a small to medium size evergreen tree. The anatomical parts belonging to* Litsea polyantha* have a long history of medicinal use among the traditional healers of Chittagong, Chittagong Hill Tracts, Sylhet and Shal forests of Gazipur, Madhupur, and Dinajpur, Bangladesh [5–9]. The bark of the plant is medicinally used as nerves and bone tonic, stimulant, analgesic, and antiseptic agent and has been given to treat diabetes, diarrhea, dysentery, and arthritis [10, 11]. Moreover, the bark* of Litsea polyantha *was reported to possess antioxidant and antidiarrheal activities [10, 12]. The leaves are traditionally used as purgative and laxative [13]. The leaves of this plant have also been reported for antimicrobial, anti-inflammatory, membrane stabilization, antiatherothrombosis, antihyperglycemic, antidiarrhoeal, antibacterial, and antifungal activity [14–17]. The root of the plant is traditionally used in pains, bruises, contusions, and fractures in animals [18]. However, the root of* Litsea polyantha *has not yet been scientifically investigated for the antioxidant, antimicrobial, cytotoxic, antihyperglycemic, and neuropharmacological effect. So, the present study was planned to identify the different chemical groups of root of* Litsea polyantha *and evaluate the aforementioned pharmacological activities.

## 2. Materials and Methods

### 2.1. Plant Material

Roots of* Litsea polyantha *were collected from Botanical Garden and Eco Park, Sitakundo, Chittagong, Bangladesh, at day time in July 2015. The plant was identified by the taxonomists of Bangladesh National Herbarium, Mirpur, Dhaka, and voucher specimen was deposited there for further reference (DACB accession number: 43173).

### 2.2. Extraction Process

At first, the collected plant parts were washed by fresh water carefully to keep them far from any kind of dust and foreign particles and dried under shed. After drying the chopped roots were grounded into fine powder. Powdered material (100 gm) was then soaked into 300 mL 96% ethanol into a tightly stoppered wide mouthed glass jar to prevent any solvent evaporation for 14 days with occasional shaking for proper mixing. After 14 days the macerated powder was filtered by Whatman filter paper and filtrate was collected. The solvent was then evaporated in a rotary evaporator at 40°C and a concentrated mass was obtained which was marked as crude extract. The yield was 1.5%.

### 2.3. Experimental Animals

Healthy Swiss Albino mice of both sexes, weighing about 20–25 gm, were used for the experiments which were purchased from the International Centre for Diarrheal Disease and Research, Bangladesh (ICDDR, B). They were housed in cages in ambient temperature with maintaining 12 hr light and 12 hr dark cycles at well ventilated animal house of Pharmacy Discipline, Khulna University, Bangladesh, for one week before the experiments with the aim of their environmental adaptation. The animals were fed with standard food (ICDDR, B formulated) and fresh water. Before commencing the experiments on these animals, clearance was taken from the Animal Ethics Committee Pharmacy Discipline, Life Science School, Khulna University, Bangladesh, and the reference number is KU/PHARM/AEC/15/006/020.

### 2.4. Chemicals

The standard drug glibenclamide (antihyperglycemic) and diazepam (CNS depressant) were purchased form Square Pharmaceutical Ltd., Bangladesh, vincristine sulphate was obtained from Beacon Pharmaceuticals Limited, Bangladesh, the glucose was purchased from GlaxoSmithKline, Bangladesh, ciprofloxacin discs (5 *μ*g/disc) were purchased from Oxoid Ltd., Basingstoke, United Kingdom, Folin Ciocalteu's reagent, quercetin, and 2,-2-diphenyl-1-picrylhydrazyl (DPPH) were procured from Sigma Chemical Co. Ltd. (St. Louis, MO, USA), and tween-80 was purchased from Loba Chemie Pvt Ltd. of India. Ethanol, methanol, gallic acid, sodium carbonate, sodium nitrite, aluminum chloride, sodium hydroxide, and dimethyl sulfoxide were obtained from Merck, Germany and all were analytical grade.

### 2.5. Phytochemical Test

The ethanolic root extract of* Litsea polyantha *were qualitatively screened with the aim of identifying the presence of different bioactive phytomolecules following the standard procedures described by Ghani, 1998 [19].

### 2.6. Antioxidant Activity Test

#### 2.6.1. Qualitative Test of Antioxidants (DPPH Free Radical Scavenging Assay)

Antioxidant potential of root extract was measured on the basis of their scavenging capacity of a stable DPPH free radical on thin layer chromatography (TLC) plate [20]. Test sample was suitably diluted and uniformly spotted on precoated silica gel TLC plates. TLC plates were developed in medium polar (CHCl_3_ : CH_3_OH = 5 : 1), polar (CHCl_3_ : CH_3_OH : H_2_O = 40 : 10 : 1), and nonpolar (*n*-Hexane : Acetone = 3 : 1) solvent systems to resolve medium polar, polar, and nonpolar components of the extract. After developing, the plates were allowed to dry in open air for a while and then sprayed with 0.02% DPPH solution in ethanol by the help of an atomizer. The bleaching of purple color of DPPH reagent to yellow color on purple background was assessed for the presence of possible antioxidant potential of the crude extract.

#### 2.6.2. Quantitative Analysis of Antioxidants (DPPH Free Radical Scavenging Assay)

The root extract of* Litsea polyantha *was dissolved in ethanol to get stock solution at 1024 *μ*g/mL concentration. Then, the stock solution was subjected to serial dilution to obtain solution at 512, 256, 128, 64, 32, 16, 8, 4, 2, and 1 *μ*g/mL concentration. Afterwards, 2 mL solution of each sample was withdrawn and 6 mL of 0.004% ethanolic solution of DPPH was added in each test tube. The mixture was shaken vigorously for 15 seconds and kept for 30 minutes at dark place at room temperature to promote reaction. The absorbance (optical density-OD) was recorded using a UV spectrophotometer for each concentration against a blank at 517 nm. The experiment was repeated twice. Ascorbic acid was taken as positive control and similarly allowed to react with DPPH and the absorbances were recorded. The average value of each concentration was used to calculate percentage of inhibition from following equation:

(1) $$\begin{array}{c} \text{\% inhibition of DPPH }=\left(\;\text{1}-\frac{\text{Sample OD}}{\text{Blank OD}}\;\right)\times 100. \end{array}$$

#### 2.6.3. Determination of Total Phenolic Content

Total phenolic content of the ethanol extract of* Litsea polyantha* was measured according to modified colorimetric Folin-Ciocalteu's method [21]. Briefly, a volume of 0.5 mL extract solution in methanol was subsequently mixed with 5 mL 10% (v/v) aqueous Folin-Ciocalteu's reagent and 4 mL of 7.5% w/v aqueous sodium carbonate. The mixture was shaken for 15 seconds and allowed to incubate at 40°C for 30 minutes. The absorbance of the mixture was then recorded using the same spectrophotometer at 765 nm wavelength against blank and compared to a standard curve obtained from gallic acid solution of different concentration in methanol. The absorbance of each concentration was measured for two times and mean value was used for further calculation. Total phenolic content value was expressed as mg of gallic acid equivalent (GAE) per gram of dry extract.

#### 2.6.4. Determination of Total Flavonoid Content

The total flavonoid content of ethanol extract of* Litsea polyantha* root was measured following aluminum trichloride colorimetric method [22]. A volume of 1 mL of a known concentration of ethanol extract was added to test tube containing 4 mL distilled water and 0.3 mL 5% (w/v) sodium nitrate solution which was allowed to stand for 5 minutes. Then, 0.3 mL 10% (w/v) aluminum chloride was added to the mixture and allowed to stand for 1 minute. Afterwards 2 mL of 1 M sodium hydroxide solution was aliquoted into the mixture and the volume of the mixture was adjusted to 10 mL by adding distilled water, shaken for 15 seconds, and allowed to react for further 30 minutes. The absorbance of the mixture was recorded at 510 nm against the blank by using the same spectrophotometer. The absorbance of each concentration was measured for two times and mean was used. The measurement was compared with a standard curve of quercetin solution in methanol of different concentration. Finally, total flavonoid content value was expressed as mg of quercetin equivalent (QE) per gram of dry extract.

#### 2.6.5. Determination of Total Tannin Content

The tannins of ethanol extract of* Litsea polyantha* were measured using the Folin-Ciocalteu phenol reagents [23]. At first, 0.1 mL of the sample extract was diluted with 7.5 mL of distilled water. Folin-Ciocalteu phenol reagent (0.5 mL) was aliquoted into test tube containing diluted solution of extract. Then, 1 mL of 35% sodium carbonate solution was added to that mixture and adjusted to 10 mL with distilled water. The mixture was shaken and allowed to stand for 30 min at room temperature. The absorbance of the mixture was recorded at 510 nm against the blank by using spectrophotometer and compared to a standard curve of prepared gallic acid solutions in methanol. Each concentration was tested in duplicate and mean absorption was taken. Total tannin content was expressed as mg of gallic acid equivalent per gram of dry extract.

### 2.7. Determination of Antimicrobial Activity by the Disc Diffusion Method

Antimicrobial activity of the ethanol extract of root of* Litsea polyantha* was evaluated by disc diffusion method [24]. Nutrient agar media were prepared by adding distilled water and sterilized in volumetric flask and cooled to 45–50°C. The sterilized agar media were poured into sterilized Petri dishes with a diameter of 120 mm and allowed to cool for few minutes to prepare the agar slants. The crude ethanolic extract of* Litsea polyantha* was applied on filter paper discs of 6 mm in diameter at concentration of 250 and 500 *μ*g/disc. After that, the impregnated discs were placed onto the agar plates containing previously inoculated gram positive and gram negative test bacteria. The gram positive bacteria included* Staphylococcus aureus* and* Streptococcus pyogenes *and gram negative bacteria included* Escherichia coli, Vibrio cholera*,* Pseudomonas aeruginosa*, and* Salmonella typhi.* The Petri dishes were kept at 4°C for 2 h and then were incubated at 37°C for 16 h. The antibacterial activity of the test agent was determined by measuring the diameter of zone of inhibition in millimeter with the help of slide calipers. Ciprofloxacin was used as positive control at the dose of 5 *μ*g/disc. The experiment was duplicated and the average zone of inhibition was used.

### 2.8. Determination of Cytotoxic Activity by Brine Shrimp Lethality Bioassay

General toxicity of the ethanolic extract of root of* Litsea polyantha *was determined by following the method developed by Meyer et al. [25]. This test was conveniently performed on brine shrimp* (Artemia Salina)*. Brine shrimp nauplii were obtained by hatching brine shrimp eggs in simulated sea water prepared by dissolving 38 gm fresh salt in 1 L distilled water. The tank water was continuously saturated by oxygen by means of an air pump. The bath temperature was maintained at 28°C with the help of an electric bulb. The brine shrimps were turned to mature nauplii within 24 h. In this test, 7 clean test tubes were taken for sample and positive control and 5 clean test tubes were taken for negative control. Stock solution of test sample was prepared by dissolving in distilled water with DMSO to obtain concentration 640 *μ*g/mL. Each test tube was accurately marked to indicate the 10 mL volume with the help of another test tube containing 10 mL sea water. Then with the help of the micropipette, 5 mL samples of each concentration, that is, 10, 20, 40, 80, 160, 320, and 640 *μ*g/mL, were transferred to 7 test tubes through serial dilution technique and adjusted to 10 mL with saline water to get final concentration of 5, 10, 20, 40, 80, 160, and 320 *μ*g/mL, respectively. Stock solution of positive control (vincristine sulphate) in concentration 20 *μ*g/mL was also subjected to serial dilution to obtain final concentration of 0.156, 0.312, 0.625, 1.25, 2.5, 5, and 10 *μ*g/mL [26]. Rest of test tubes contained only DMSO in 10 mL sea water as negative control. 10 living nauplii were transferred to every test tube with the help of a Pasteur pipette. The concentration of DMSO in each test tube did not exceed 10 *μ*L/mL. After 24 h, numbers of living nauplii were counted using magnifying glass and noted carefully. The experiment was performed twice to minimize error.

### 2.9. Determination of Antihyperglycemic Activity by Oral Glucose Tolerance Test (OGTT)

Antihyperglycemic activity of ethanol extract of* Litsea polyantha *root was determined by following method described by Djilani et al. [27] with few modifications. In brief, overnight fasted mice of both sexes were divided into four groups of five mice each. The groups were referred as negative control group which were orally given vehicle (1% tween 80 in water at dose of 10 mL/kg body weight), positive control group which were treated orally with a standard hypoglycemic drug, glibenclamide at the dose of 10 mg/kg body weight, and test groups 1 and 2 which were orally treated with the plant extract at two different doses of 250 and 500 mg/kg body weight, respectively. At the onset, the blood glucose level of all mice was recorded with the help of a glucometer by punching the tail with a sterile needle. Then the extract, positive control, and negative control solution were given orally by means of a feeding needle maintaining the aforementioned dose level(s). After 30 minutes, all mice were orally given glucose at dose of 2 gm/kg body weight by means of a feeding needle. Blood glucose levels were again measured using a glucometer at 30 minutes, 90 minutes, and 120 minutes of administration of glucose with the help of same glucometer in terms of mmol/L. The experiment was duplicated to minimize the statistical error.

### 2.10. Determination of Neuropharmacological Activity

#### 2.10.1. Open Field Test

The neuropharmacological activity of ethanol extract of roots of* Litsea polyantha *was determined following the method described by Shilpi et al. [28]. In this test, the mice were randomly divided into four groups of five members each. The four groups were marked as negative control, positive control, and test groups (1 and 2), respectively. The negative control group was treated with vehicle (1% tween 80 in water) at dose of 10 mL/kg body weight, positive control group was given a standard central nervous system (CNS) depressant drug diazepam at a dose of 1 mg/kg body weight, and test groups were given the plant extract at two different doses of 250 and 500 mg/kg body weight. All treatments were performed orally with the help of clean feeding needle. The floor of open field apparatus was surrounded by wall of 40 cm height to avoid mice escaping and splitting into a series of square grids (100 cm × 100 cm). In this apparatus, the four corners' squares of the field were painted with black color and rest of the squares were painted with white color. On different time intervals (0, 30, 60, 90, 120, and 180 min) of respective treatment, the number of squares visited by the treated animals for 3 min was recorded. During experiment silent environment was strictly maintained.

#### 2.10.2. Hole Cross Test

The method followed by Uddin et al. [29] was adopted to evaluate the neuropharmacological activity of ethanol extract of roots of* Litsea polyantha. *In hole cross test, mice of either sex were divided into four groups of five members each. Negative control group received 1% tween 80 in water at dose of 10 mL/kg body weight, positive control group received diazepam at dose of 1 mg/kg body weight, and test groups received the extract at two different doses of 250 and 500 mg/kg body weight. All doses were given orally with the help of a clean feeding needle. The hole cross apparatus had two chambers partitioned by a wall of 7.5 cm height with a hole of 3 cm diameter at bottom of the partition. In the apparatus, one chamber exhibited dark environment and another exhibited light environment. On different time intervals (0, 30, 60, 90, 120, and 180 min) of respective treatment, the number of crosses by the animal through the hole from one chamber to another for 3 min was recorded. During experiment silent environment was strictly maintained.

#### 2.10.3. Hole Board Test

The method described by File and Wardill [30] was followed to assay neuropharmacological activity of ethanol extract of roots of* Litsea polyantha*. In this test, the mice were randomly divided into four groups which referred as negative control, positive control and test groups containing five mice each, where negative control group was given vehicle as 1% tween 80 in water at dose of 10 mL/kg body weight, positive control group was given standard drug as diazepam at a dose of 1 mg/kg body weight and test groups were given extract at two different doses of 250 and 500 mg/kg body weight. All doses were given orally with the help of a clean feeding needle. The floor of hole board test apparatus had been evenly distributed into 16 holes surrounding with wall of 40 cm height to avoid escaping of mice. On different time intervals (0, 30, 60, 90, 120, and 180 min) of respective treatment, the total amount of head dipping of mice in the hole for 3 min was recorded. During experiment silent environment was strictly maintained.

### 2.11. Statistical Analysis

Statistical analysis was performed using Microsoft excel and Graph Pad prism version 5.0 (GraphPad software Inc., San Diego, CA). Experimental values were expressed as mean (*n* = 5) ± standard error of mean (SEM). Two-way ANOVA followed by Bonferroni's test was used for statistical comparison. Experimental values were considered statistically significant when *p* < 0.05 in all cases. The LD_50_ value in brine shrimp lethality bioassay was determined by using Probit analysis software (Ldp line software, USA).

## 3. Results

### 3.1. Phytochemical Group Test

The tests carried out on ethanol extract of roots of* Litsea polyantha *indicated the presence of diverse secondary metabolites, namely, reducing sugar, combined reducing sugar, tannins, flavonoids, alkaloids, terpenoids, and phenol.

### 3.2. Antioxidant Activity Test

#### 3.2.1. Determination of* In Vitro* Antioxidant Activity (DPPH Free Radical Scavenging Property)

**Figure 1:**
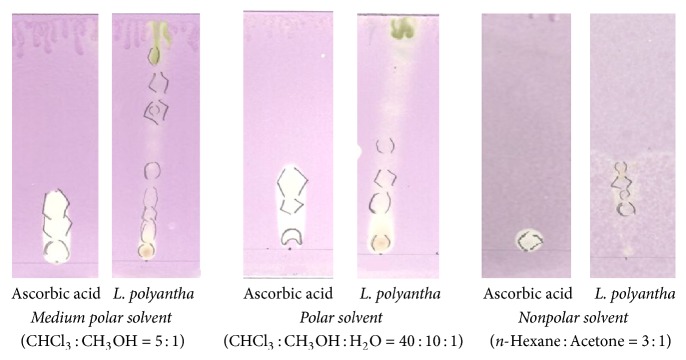
Comparison of TLC plate of* Litsea polyantha *with standard (ascorbic acid) after applying 0.02% DPPH.

In qualitative antioxidant activity assay, root extract of* Litsea polyantha *bleached DPPH reagent on TLC plate from its deep purple color to yellow color on purple background which revealed the presence of antioxidant components in the extract (Figure 1).

**Figure 2:**
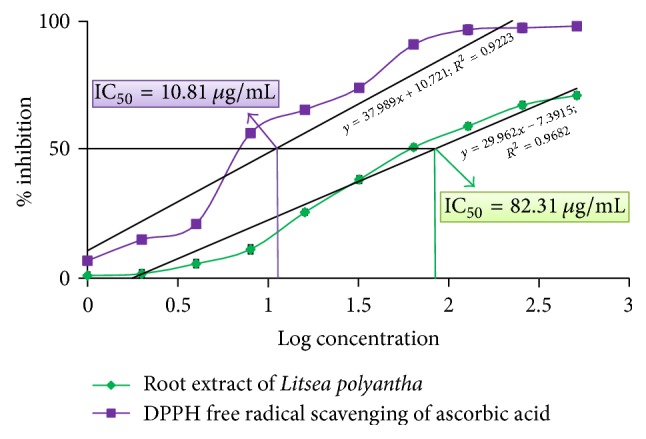
DPPH scavenging activity of root extract of* Litsea polyantha*.

On the other hand, in quantitative antioxidant activity assay, root extract of* Litsea polyantha *exhibited antioxidant activity in concentration dependent manner (IC_50_ = 82.31 *μ*g/mL) against DPPH free radical which is comparable to that of ascorbic acid (IC_50_ = 10.81 *μ*g/mL) (Figure 2; see Supplementary Table 1 in Supplementary Material available online at https://doi.org/10.1155/2017/3701349)

#### 3.2.2. Determination of Total Phenolic Content

**Figure 3:**
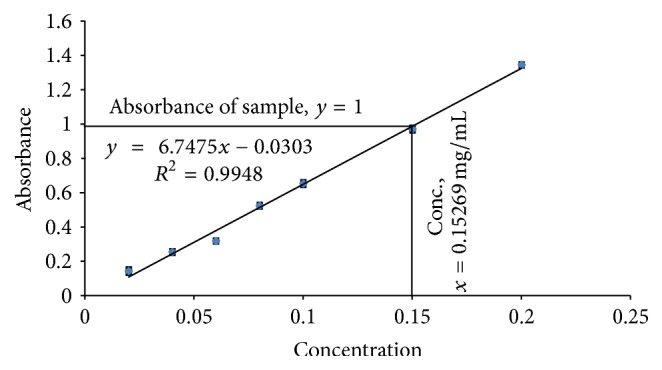
Determination of total phenolic content from gallic acid calibration curve.

The total phenolic content of crude extract was calculated by using linear regression equation, *y* = 6.7475*x* − 0.0303 (*R*^2^ = 0.9948), obtained from a standard gallic acid calibration curve (Figure 3 and Supplementary Table 2), and found to be 152.69 mg GAE/g dry extract.

#### 3.2.3. Determination of Total Flavonoid Content

**Figure 4:**
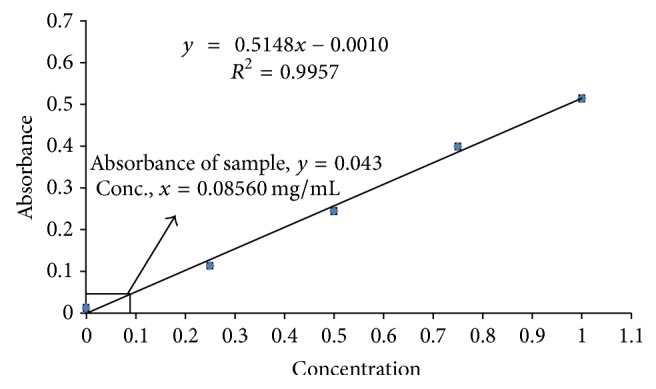
Determination of total flavonoid content using quercetin calibration curve.

The total flavonoid content of crude extract was calculated by using linear regression equation, *y* = 0.5148*x* − 0.0010 (*R*^2^ = 0.9957), obtained from a standard quercetin calibration curve (Figure 4 and Supplementary Table 3), and found to be 85.60 mg QE/g dry extract.

#### 3.2.4. Determination of Total Tannin Content

**Figure 5:**
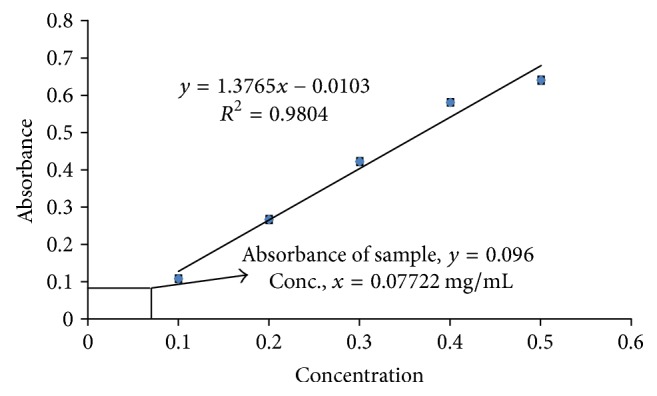
Determination of total tannin content using gallic acid calibration curve.

Total tannin content of root extract of* Litsea polyantha* was calculated by using linear regression equation, *y* = 1.3765*x* − 0.0103 (*R*^2^ = 0.9804), obtained from a standard gallic acid calibration curve (Figure 5 and Supplementary Table 4), and found to be 77.22 mg GAE/g of dried plant extract.

### 3.3. Determination of Antimicrobial Activity by the Disc Diffusion Method

**Figure 6:**
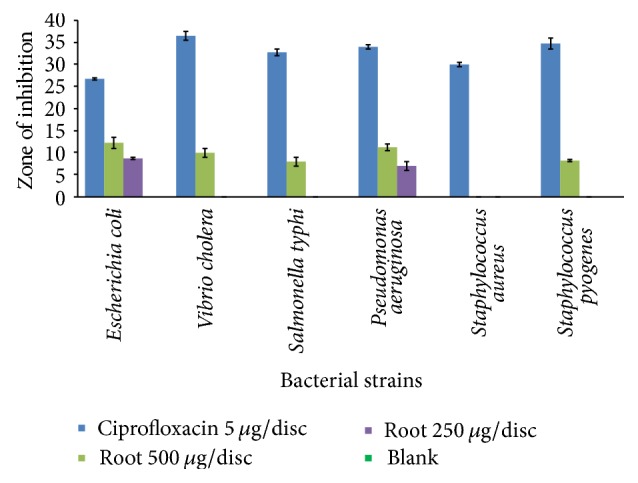
Diagram of zone of inhibition ± SEM of root extract against 6 bacterial strains.

The extract of* Litsea polyantha* root exhibited mild to moderate antibacterial potential against six different species of bacteria compared to control. The root extract at the dose of 250 *μ*g/disc exhibited zone of inhibition against* Escherichia coli *(8.75 ± 0.25 mm) and* Pseudomonas aeruginosa *(7 ± 1 mm) but displayed no zone of inhibition against* Vibrio cholera*,* Salmonella typhi*,* Staphylococcus aureus,* and* Staphylococcus pyogenes*, whereas, at the dose of 500 *μ*g/disc, the test sample exhibited zone of inhibition against* Escherichia coli* (12.25 ± 1.25 mm),* Vibrio cholera* (10 ± 1 mm),* Salmonella typhi* (8 ± 1 mm),* Pseudomonas aeruginosa *(11.25 ± 0.75 mm), and* Staphylococcus pyogenes *(8.25 ± 0.25 mm) but displayed no zone of inhibition against* Staphylococcus aureus* (Figure 6 and Supplementary Table 9).

### 3.4. Determination of Cytotoxic Activity by Brine Shrimp Lethality Bioassay

**Figure 7:**
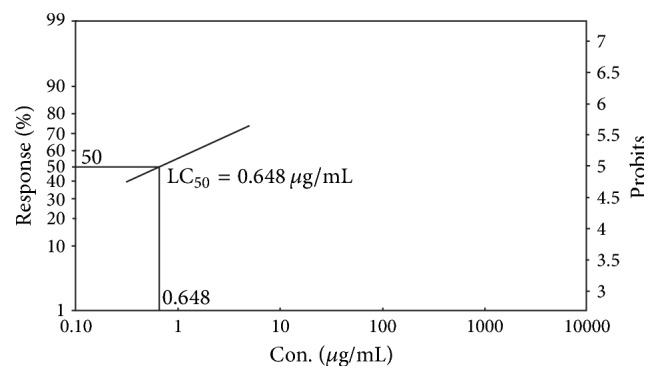
Determination of LC_50_ for the standard (vincristine sulphate) by Ldp line software.

**Figure 8:**
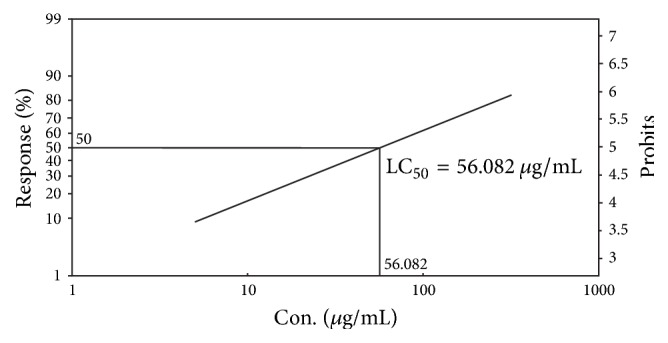
Determination of LC_50_ of ethanolic extract of* Litsea polyantha* roots by Ldp line software.

The ethanolic extract of root of* Litsea polyantha*and the positive control vincristine sulphate showed brine shrimp lethality in dose-dependent manner and exhibited approximate linear correlation between the concentration andpercent (%) of mortality. The median lethal concentration as LC_50_ expressed in terms of *μ*g/mL was ascertained by means of Probit analysis software to evaluate the toxic potentiality of the crude ethanolic extract of* Litsea polyantha*. The LC_50_ for positive control (vincristine sulphate) and crude extract were found to be 0.648 *μ*g/mL and 56.082 *μ*g/mL, respectively (Figures 7 and 8).

### 3.5. Determination of Antihyperglycemic Activity by Oral Glucose Tolerance

The root extract exhibited distinct improvements in glucose tolerance at two different doses of 250 and 500 mg/kg body weight compared to negative control (*p* < 0.05, *p* < 0.01, and *p* < 0.001). The crude extracts demonstrated highest blood glucose lowering potentiality in glucose-loaded mice at the point of 120 min at both aforementioned doses (Table 1).

### 3.6. Determination of Neuropharmacological Activity

#### 3.6.1. Open Field Test

The root extract exhibited appreciable reduction in number of squares traveled by the mice at two different doses of 250 and 500 mg/kg body weight compared to negative control (*p* < 0.01 and *p* < 0.001). The reduction of locomotors was manifested at 30 min and persisted up to 90 min, demonstrating maximum decrease of locomotors activity (Table 2).

#### 3.6.2. Hole Cross Test

The results (Table 3) of neuropharmacological effect of ethanolic root extract of* Litsea polyantha* indicated that the crude extract remarkably decreased the numbers of holes crossed by mice from one chamber to the next chamber compared to negative control (*p* < 0.05, *p* < 0.01, and *p* < 0.001) at two different doses of 250 and 500 mg/kg body weight. However, in the case of crude extract the effect could be observable since 2nd observation (at 30 min) and continued till 5th (at 120 min) observation, but in the case of standard drug diazepam the effective period was from 2nd to 4th observation in the study period.

#### 3.6.3. Hole Board Test

The crude extracts at doses of 250 and 500 mg/kg body weight exhibited moderate reduction in head dipping compared to negative control (*p* < 0.05, *p* < 0.01, and *p* < 0.001). Crude extract demonstrated its potentiality from 30 min and continued to 90 min with maximum effect whereas for standard drug the effect was continued till 120 minutes (Table 4).

## 4. Discussion

Both the quantitative and qualitative assay* (in vitro)* have been performed to screen the antioxidant potentiality of ethanolic extract of* Litsea polyantha* root with the aim of rapid screening of substances having antioxidant potentiality. Free radicals containing one or more unshared pair electrons are known to be responsible for many deadly pathological conditions in human body such as cancers, cardiovascular diseases, inflammatory diseases, respiratory diseases, diabetes mellitus, cataract, male infertility as well as aging process [31]. The antioxidant compounds exhibit their potentiality either by scavenging the reactive oxygen species or by protecting antioxidant defense mechanisms of body [32]. In TLC based qualitative antioxidant assay using DPPH, the bleaching of purple color of DPPH reagent to yellow color on purple background substantiated the extract's antioxidant activity (Figure 1) [20]. It might be due to supply of either hydrogen atoms or electron to DPPH reagent which is common mechanism of inhibition of lipid peroxidation from free radical [33, 34]. Quantitatively, the degree of decolonization of DPPH is proportional to the concentration and potency of the antioxidants. A decrease in the absorbance of the reaction mixture substantiated free radical scavenging activity of the compound under test [35]. The absorbance of DPPH solution with crude ethanolic extract of root of* Litsea polyantha* was found to be decreased with increased concentration which ascertained the free radical scavenging activity of the test extract (Figure 2). This antioxidant potentiality of plant extract may be attributed due to the presence of flavonoid and tannin which are phenolic in nature. Typically the polyphenolic compounds react through donation of hydrogen and neutralization of free radical [22]. So, we also quantified total phenolic content (Figure 3), total flavonoid (Figure 4) content, and total tannin (Figure 5) content where total phenolic content is quite higher than flavonoids and tannin which may be the possible reason for the extract's moderate antioxidant activity. Further, sophisticated experiment is necessary to identify responsible constituents for antioxidant property of* Litsea polyantha* root and characterize its* in vivo* antioxidant potentiality.

Disc diffusion assay was performed to assess the crude extract's antimicrobial activity against six bacterial strains. The result demonstrated (Figure 6) that the crude extract possess antimicrobial activity in comparison with standard drug ciprofloxacin. This antimicrobial activity of the crude extract might be attributed due to the presence of flavonoid, tannin, and alkaloid [36]. Flavonoid exhibited their antimicrobial activity either by complexing with bacterial cell wall or binding to adhesins [36]. Tannin exhibited their antimicrobial activity either by binding to proteins or inhibiting enzyme or disrupting bacterial cell membrane [36]. Alkaloid exhibited their antimicrobial activity by intercalation into cell wall and/or DNA [36]. Further, cellular investigation is needed to identify the compound(s) and their respective mechanism(s) of such inhibitory activity.

The brine shrimp lethality bioassay is a convenient, economic, and safe tool to determine bioactivity of synthetic compounds as well as crude extract [37, 38]. Though brine shrimp lethality is not any specific pharmacological activity testing protocol, it correlates reasonably well between cytotoxicity and anticancer properties [39]. The percent mortality of brine shrimp nauplii for the crude extract of* Litsea polyantha* was in concentration dependent manner in comparison with standard drug vincristine sulphate (Figure 7). The crude extract was seen to be quite toxic for brine shrimp nauplii having LC_50_ lower than 250 *μ*g/mL (approx. 56.082 *μ*g/mL, shown in Figure 8). It has been previously reported that any crude extract having LC_50_ lower than 250 *μ*g/mL might be a potential source of diverse bioactive constituent(s) especially as anticancer agents, antimalarial drugs, insecticidal, and so forth [40, 41]. Among various secondary metabolites of plants, terpenoids, flavonoids, tannins, and alkaloids possess cytotoxic or anticancer activity [42]. Flavonoids exhibited their cytotoxic or anticancer activity by inhibition of DNA topoisomerase I/II activity, decrease of reactive oxygen species (ROS), modulation of signaling pathways, downregulation of nuclear transcription factor kappa B (NF-*κ*B), activation of endonuclease, and suppression of Mcl-1 protein [43]. Tannins exert their cytotoxicity by appeasing the production of enzyme required for cancer cell line growth [42]. Terpenoids, a large class of natural products, have wide range of cytotoxic or anticancer activity such as DNA minor groove binder and inhibition of DNA topoisomerases I and II [44]. Alkaloids are the largest group of compounds and have diverse way of showing anticancer activity such as inducing cell cycle arrest and autophagy, binding avidly with tubulin, and directly interacting with glutathione [45]. The standard drug vincristine itself is an alkaloidal drug. Moreover, at our tested doses (250 and 500 mg/kg) the experimental mice did not show any kind of toxic symptoms. So, it can be apparently said that the crude extract may not be toxic for mammalian cell. More precise study is needed to judge cytotoxicity activity of* Litsea polyantha *root and identify the possible compound(s) and mechanism(s) of action.

In the study of antihyperglycemic activity on nondiabetic mice by executing oral glucose tolerance test, the extract displayed statistically significant (*p* < 0.05, *p* < 0.01, and *p* < 0.001) blood glucose lowering activity at two different doses of 250 and 500 mg/kg body weight (Table 1). The observed reduction of blood glucose level by the extract might be imposed to either of following mechanisms or combination of the mechanisms like potentiating the pancreatic secretion of insulin or increasing the glucose uptake or interfering with the intestinal glucose absorption in the gut or stimulating peripheral glucose uptake [46–48]. The alkaloids, flavonoids, and tannins present in this plant may be responsible for the observed antihyperglycemic effects [49, 50]. As it was a preliminary study, further, more precise investigation should be conducted to demonstrate possible compound(s) and their cellular activity.

The neuropharmacological activity of test extract was performed using three different test models. Statistically significant (*p* < 0.05, *p* < 0.01, and *p* < 0.001) decreased locomotor activity in experimental mice was observed in both open field test model and hole cross test model at two different doses (250 and 500 mg/kg body weight) compared to negative control (Tables 2 and 3). The observed effect was fairly similar to the consequence of standard sedative drug, diazepam. So, it could be said that extract showed depressant activity on mice. On the other hand, through the hole board test model the exploratory behavior of mice was investigated. The crude extract significantly decreased (*p* < 0.05, *p* < 0.01, and *p* < 0.001) amount of head dipping by experimental mice with a time dependent manner at the two different doses of 250 and 500 mg/kg body weight compared to negative control (Table 4). The plant extract may exhibit depressant effect following either of two known mechanisms: positive allosteric modulators of gamma amino butyric acid (GABA) receptors or increase in the amount of GABA which is an established neurotransmitter [51, 52]. It has been reported that plant extract containing alkaloids, flavonoids, tannin, and terpenoids possesses CNS depressant [53–57]. The presence of afore-cited secondary metabolites might be responsible for the CNS depressant effect of crude extract of* Litsea polyantha* root. So, further phytochemical isolation initiative is crucial to ensure the exact mechanism(s) and compound(s) to conclude such CNS depressant effect.

Though this study reports preliminary investigation about* Litsea polyantha* root's phytoconstituents and their possible bioactivities, it may be of great interest for the phytochemists for further isolation and characterization of bioactive constituents from this indigenous plant. The active phytoconstituents identified by these means may contribute to discovering new drug leads.

## 5. Conclusion

The present study based on diverse methodological approaches suggests that the ethanolic crude extract of* Litsea polyantha* is fortified with antioxidant, antimicrobial, cytotoxic, antihyperglycemic, and neuropharmacological modulating compounds. Further exploration for isolation and characterization of such compounds may contribute to drug discovery using natural sources.

## Supplementary Material

Supplemental Table-1: DPPH Free Radical Scavenging Assay.
Supplemental Table-2: Total Phenolic Content.
Supplemental Table-3: Total Flavonoid content.
Supplemental Table-4: Total Tannin content.
Supplemental Table-5: ANOVA results of the effects of ethanol extract of roots of *Litsea polyantha* on glucose-loaded mice.
Supplemental Table- 6: ANOVA results of neuropharmacological effect of ethanol extract of *Litsea polyantha* on open field test.
Supplemental Table- 7: ANOVA results of neuropharmacological effect of ethanol extract of *Litsea polyantha* on hole Cross test.
Supplemental Table-8: ANOVA results of neuropharmacological effect of ethanol extract of *Litsea polyantha* on hole board test.
Supplemental Table-9: Diagram of zone of inhibition ± SEM of root extract against 6 bacterial strains.

## Figures and Tables

**Table 1:** Effects of ethanol extract of roots of *Litsea polyantha* on glucose-loaded mice.

Treatment (oral) group	Blood glucose level (mmol/L)			
	Fasting state (before treatment)	30 min	90 min	120 min
Negative control	5.70 ± 0.24	13.66 ± 0.71	8.74 ± 0.35	7.16 ± 0.24
Positive control (glibenclamide)	5.96 ± 0.12	6.38 ± 0.19^c^	3.88 ± 0.21^c^	2.96 ± 0.081^c^
Root 250 mg/kg	4.38 ± 0.33	12.34 ± 0.72	7.16 ± 0.39^a^	5.28 ± 0.26^b^
Root 500 mg/kg	4.68 ± 0.35	11.32 ± 0.47^c^	5.48 ± 0.33^c^	4.94 ± 0.26^c^

^a^ *p* < 0.05, ^b^*p* < 0.01, and ^c^*p* < 0.001 when compared with negative control. The significance level for individual experiment period has been shown in Supplementary Table 5.

**Table 2:** Neuropharmacological effect of ethanol extract of *Litsea polyantha *on open field test.

Treatment (oral) group	Number of squares crossed by the mice					
	0 min	30 min	60 min	90 min	120 min	180 min
Negative control	117.2 ± 3.89	89.00 ± 1.95	76.80 ± 1.50	71.0 ± 2.07	79.60 ± 1.88	82.40 ± 1.33
Positive control (diazepam)	125.60 ± 2.56	37.0 ± 1.41^c^	31.0 ± 0.84^c^	28.0 ± 0.89^c^	32.4 ± 0.60^c^	29.8 ± 0.80^c^
Root (250 mg/kg)	119.0 ± 2.81	68.8 ± 2.03^c^	54.6 ± 1.63^c^	50.6 ± 2.5^c^	61.2 ± 2.6^c^	70.20 ± 2.67^b^
Root (500 mg/kg)	120.60 ± 3.8	59.2 ± 0.80^c^	48.8 ± 2.22^c^	44.8 ± 2.22^c^	57.4 ± 3.4^c^	65.2 ± 0.66^c^

^b^ *p* < 0.01 and ^c^*p* < 0.001 when compared with negative control. The significance level for individual experiment period has been shown in Supplementary Table 6.

**Table 3:** Neuropharmacological effect of ethanolic extract of *Litsea polyantha* on hole cross test.

Treatment (oral) group	Number of holes crossed by the mice					
	0 min	30 min	60 min	90 min	120 min	180 min
Negative control	10.2 ± 0.80	8.6 ± 0.51	8.2 ± 0.58	7.6 ± 0.51	6.6 ± 0.5	8.6 ± 0.92
Positive control (diazepam)	9.4 ± 0.51	2.4 ± 0.4^c^	1.8 ± 0.37^c^	1.4 ± 0.51^c^	2.0 ± 0.70^c^	2.2 ± 0.37^c^
Root 250 mg/kg	10.8 ± 0.37	8.0 ± 0.55	7.8 ± 0.37	7.0 ± 0.32	6.0 ± 0.32	7.8 ± 0.37
Root 500 mg/kg	10.2 ± 0.37	7.6 ± 0.51	6.4 ± 0.51	5.2 ± 0.37^b^	5.0 ± 0.32	6.4 ± 0.24^a^

^a^ *p* < 0.05, ^b^*p* < 0.01, and ^c^*p* < 0.001 when compared with negative control. The significance level for individual experiment period has been shown in Supplementary Table 7.

**Table 4:** Neuropharmacological effect of ethanolic extract of *Litsea polyantha* on hole board test.

Treatment (oral) group	Amount of head dipping by the mice					
	0 min	30 min	60 min	90 min	120 min	180 min
Negative control	17 ± 0.71	22 ± 1.14	18 ± 0.71	21.4 ± 0.75	25 ± 0.71	29.2 ± 0.66
Positive control (diazepam)	19 ± 0.71	11.6 ± 0.51^c^	8.4 ± 0.51^c^	6 ± 0.45^c^	3.2 ± 0.37^c^	7 ± 0.45^c^
Root 250 mg/kg	19.8 ± 0.86^a^	18.4 ± 0.51^c^	15.4 ± 0.51^a^	14.2 ± 0.37^c^	19 ± 0.71^c^	20.6 ± 0.57^c^
Root 500 mg/kg	19.2 ± 0.37	17.4 ± 0.51^c^	15 ± 0.71^b^	14.8 ± 0.58^c^	18 ± 0.71^c^	20.4 ± 0.93^c^

^a^ *p* < 0.05, ^b^*p* < 0.01, and ^c^*p* < 0.001 when compared with negative control. The significance level for individual experiment period has been shown in Supplementary Table 8.

## References

[1] De Pasquale A. (1984). Pharmacognosy: The oldest modern science. *Journal of Ethnopharmacology*.

[2] Fabricant D. S., Farnsworth N. R. (2001). The value of plants used in traditional medicine for drug discovery. *Environmental Health Perspectives*.

[3] Rates S. M. K. (2001). Plants as source of drugs. *Toxicon*.

[4] Krause J., Tobin G. (2013). Discovery, development, and regulation of natural products. *Using Old Solutions to New Problems-Natural Drug Discovery in the 21St Century*.

[5] Sajib N. H., Uddin S. B. (2015). Ethnomedicinal study of plants in Hathazari, Chittagong, Bangladesh. *Pertanika Journal of Tropical Agricultural Science*.

[6] Morshed A. J. M., Nandni N. C. (2012). Indigenous medicinal plants used by the tribal healers of chittagong hill tracts to treat diarrhoea and dysentery. *Hamdard Medicus*.

[7] Rana M. P., Sohel M. S. I., Akhter S., Islam M. J. (2010). Ethno-medicinal plants use by the Manipuri tribal community in Bangladesh. *Journal of Forestry Research*.

[8] Kamal Z., Bairage J. J., Moniruzzaman P. R. D. (2014). Folk medicinal uses of some plants in Tangail district, Bangladesh. *World Journal of Pharmacy and Pharmaceutical Sciences*.

[9] Dr. Shaikh Bokhtear Uddin, "Medicinal Plants of Bangladesh," http://www.mpbd.info/plants/litsea-monopetala.php

[10] Arfan M., Amin H., Kosinska A., Karamac M., Amarowicz R. (2008). Antioxidant activity of phenolic fractions of Litsea monopetala [Persimon-leaved litsea] bark extract. *Polish Journal of Food and Nutrition Sciences*.

[11] Yeasmin M., Karmaker S., Hossain M. S. (2015). A case study of an urban garo tribal medicinal practitioner in Mymensingh district, Bangladesh. *World Journal of Pharmacy and Pharmaceutical Sciences*.

[12] Poonia B. S., Sasmal D., Mazumdar P. M. (2007). Anti-diarrheal activity of methanol extract of Litsea polyantha bark in mice. *Fitoterapia*.

[13] Bhattacharya S. B., Sarkar K. K., Banerji N. (1984). Some structural aspects of the mucilage isolated from the leaves of Litsea polyantha. *Carbohydrate Research*.

[14] Ahmmad A., Islam M. T., Sultana I. (2012). Pharmacological and phytochemical screening of ethanol extract of Litsea monopetala (Roxb.) Pers. *International Organization of Scientific Research Journal of Pharmacy*.

[15] Hasan H., Azad M. S. A., Islam M. Z. (2014). Antihyperglycemic activity of methanolic extract of Litsea monopetala (Roxb.) Pers. leaves. *Advances in Natural and Applied Sciences*.

[16] Nasrin F., Hakim M. L. (2015). In vivo antidiarrheal study of ethanolic extracts of Mikania cordata and Litsea monopetala leaves. *Bangladesh Journal of Pharmacology*.

[17] Hasan M., Iqbal M., Uddin M. (2016). Antibacterial and antifungal activity of litsea monopetala leaves on selected pathogenic strains. *European Journal of Medicinal Plants*.

[18] Ghosh M., Sinha B. N., Seijas J. A., Vázquez-Tato M. P., Feás X. Optimization process for increasing the yield of crude alkaloid from litsea polyantha juss.

[19] Ghani A. (1998). Medicinal plants of Bangladesh: chemical constituents and uses. *Asiatic society of Bangladesh, Dhaka*.

[20] Biswas N. N., Saha S., Ali M. K. (2014). Antioxidant, antimicrobial, cytotoxic and analgesic activities of ethanolic extract of Mentha arvensis L.. *Asian Pacific Journal of Tropical Biomedicine*.

[21] Wolfe K., Wu X., Liu R. H. (2003). Antioxidant activity of apple peels. *Journal of Agricultural and Food Chemistry*.

[22] Zilani M. N., Islam M. A., Khushi S. S., Shilpi J. A., Rahman M. M., Hossain M. G. (2016). Analgesic and antioxidant activities of *Colocasia fallax*. *Oriental Pharmacy and Experimental Medicine*.

[23] Tamilselvi N., Krishnamoorthy P., Dhamotharan R., Arumugam P., Sagadevan E. (2012). Analysis of total phenols, total tannins and screening of phytocomponents in indigofera aspalathoides (shivanar vembu) Vahl EX DC. *Journal of Chemical and Pharmaceutical Research*.

[24] Ahmed F., Das P. K., Islam M. A., Rahman K. M., Rahman M. M., Selim M. S. T. (2003). Antibacterial activity of Cordyline terminalis. Kunth. leaves. *Journal of Medical Science*.

[25] Meyer B. N., Ferrigni N. R., Putnam J. E., Jacobsen L. B., Nichols D. E., McLaughlin J. L. (1982). Brine shrimp: a convenient general bioassay for active plant constituents. *Planta Medica*.

[26] Rahman M. A., Sultana R., Bin Emran T. (2013). Effects of organic extracts of six Bangladeshi plants on *in vitro* thrombolysis and cytotoxicity. *BMC Complementary and Alternative Medicine*.

[27] Djilani A., Toudert N., Djilani S. (2011). Evaluation of the hypoglycemic effect and antioxidant activity of methanol extract of Ampelodesma mauritanica roots. *Life Sciences and Medicine Research*.

[28] Shilpi J. A., Uddin S. J., Rouf R., Billah M. (2004). Central nervous system depressant activity of Diospyros peregrina bark. *Oriental Pharmacy and Experimental Medicine*.

[29] Uddin S. J., Shilpi J. A., Rahman M. T., Ferdous M., Rouf R., Sarker S. D. (2006). Assessment of neuropharmacological activities of Pandanus foetidus (Pandanaceae) in mice. *Die Pharmazie-An International Journal of Pharmaceutical Sciences*.

[30] File S. E., Wardill A. G. (1975). Validity of head-dipping as a measure of exploration in a modified hole-board. *Psychopharmacology*.

[31] Rahman T., Hosen I., Islam M. M. T., Shekhar H. U. (2012). Oxidative stress and human health. *Advances in Bioscience and Biotechnology*.

[32] Islam E., Islam R., Rahman A. A. (2013). Estimation of total phenol and in vitro antioxidant activity of Albizia procera leaves. *BMC Research Notes*.

[33] Malki F., Touati A., Moulay S., Baltas M. (2016). Antioxidant and antimicrobial activities of two amidine derivatives. *Mediterranean Journal of Biosciences*.

[34] Bajpai V. K., Sharma A., Kang S. C., Baek K.-H. (2014). Antioxidant, lipid peroxidation inhibition and free radical scavenging efficacy of a diterpenoid compound sugiol isolated from Metasequoia glyptostroboides. *Asian Pacific Journal of Tropical Medicine*.

[35] Krishnaiah D., Sarbatly R., Nithyanandam R. (2011). A review of the antioxidant potential of medicinal plant species. *Food and Bioproducts Processing*.

[36] Cowan M. M. (1999). Plant products as antimicrobial agents. *Clinical Microbiology Reviews*.

[37] De Almeida P. A., Da Silva T. M. S., Echevarria A. (2002). Mesoionic 5-alkyl-1,3-dithiolium-4-thiolates: synthesis and brine shrimp toxicity. *Heterocyclic Communications*.

[38] Krishnaraju A. V., Rao T. V., Sundararaju D., Vanisree M., Tsay H.-S., Subbaraju G. V. (2005). Assessment of bioactivity of indian medicinal plants using brine shrimp (artemia salina) lethality assay. *International Journal of Applied Science and Engineering*.

[39] Hasan M. S., Ahmed M. I., Mondal S. (2006). Antioxidant, antinociceptive activity and general toxicity study of Dendrophthoe falcata and isolation of quercitrin as the major component. *Oriental Pharmacy and Experimental Medicine*.

[40] Rieser M. J., Gu Z.-M., Fang X.-P., Zeng L., Wood K. V., McLaughlin J. L. (1996). Five novel mono-tetrahydrofuran ring acetogenins from the seeds of *Annona muricata*. *Journal of Natural Products*.

[41] Dusen S., Aydin C., Gul H. Y., Ozay C., Dusen O., Mammadov R. (2016). In vitro cytotoxic activities of cyclamen L. (primulaceae) ethanol extracts from turkey, fresenius envir. *Fresenius Environmental Bulletin*.

[42] Musa A. A. (2012). Cytotoxicity activity and phytochemical screening of Cochlospermum tinctorium perr ex A. Rich rhizome. *Journal of Applied Pharmaceutical Science*.

[43] Chahar M. K., Sharma N., Dobhal M. P., Joshi Y. C. (2011). Flavonoids: a versatile source of anticancer drugs. *Pharmacognosy Reviews*.

[44] Huang M., Lu J., Huang M., Bao J., Chen X., Wang Y. (2012). Terpenoids: Natural products for cancer therapy. *Expert Opinion on Investigational Drugs*.

[45] Lu J., Bao J. L., Chen X. P., Huang M., Wang Y. (2012). Alkaloids isolated from natural herbs as the anticancer agents. *Evidence-Based Complementary and Alternative Medicine*.

[46] Nyunaï N., Njikam N., Abdennebi E. H., Mbafor J. T., Lamnaouer D. (2009). Hypoglycaemic and antihyperglycaemic activity of *Ageratum conyzoides* L. in rats. *African Journal of Traditional, Complementary and Alternative Medicines*.

[47] Bhowmik A., Khan L. A., Akhter M., Rokeya B. (2009). Studies on the antidiabetic effects of Mangifera indica stem-barks and leaves on nondiabetic, type 1 and type 2 diabetic model rats. *Bangladesh Journal of Pharmacology*.

[48] DeFronzo R. A., Ferrannini E., Sato Y., Felig P., Wahren J. (1981). Synergistic interaction between exercise and insulin on peripheral glucose uptake. *Journal of Clinical Investigation*.

[49] Jung M., Park M., Lee H. C., Kan Y., Kang E. S., Kim S. K. (2006). Antidiabetic agents from medicinal plants. *Current Medicinal Chemistry*.

[50] Suba V., Murugesan T., Arunachalam G., Mandal S. C., Saha B. P. (2004). Anti-diabetic potential of Barleria lupulina extract in rats. *Phytomedicine*.

[51] Foster A. C., Kemp J. A. (2006). Glutamate- and GABA-based CNS therapeutics. *Current Opinion in Pharmacology*.

[52] Chapouthier G., Venault P. (2001). A pharmacological link between epilepsy and anxiety?. *Trends in Pharmacological Sciences*.

[53] Vohora S. B., Kumar I., Khan M. S. Y. (1984). Effect of alkaloids of Solanvm melongena on the central nervous system. *Journal of Ethnopharmacology*.

[54] Fernández S., Wasowski C., Paladini A. C., Marder M. (2004). Sedative and sleep-enhancing properties of linarin, a flavonoid-isolated from *Valeriana officinalis*. *Pharmacology Biochemistry & Behavior*.

[55] Kahnberg P., Lager E., Rosenberg C. (2002). Refinement and evaluation of a pharmacophore model for flavone derivatives binding to the benzodiazepine site of the GABAA receptor. *Journal of Medicinal Chemistry*.

[56] Takahashi R. N., De Lima T. C. M., Morato G. S. (1986). Pharmacological actions of tannic acid; II. Evaluation of CNS activity in animals. *Planta Medica*.

[57] Meckes M., Calzada F., Tortoriello J., Gonzalez J. L., Martínez M. (1996). Terpenoids isolated from Psidium guajava hexane extract with depressant activity on central nervous system. *Phytotherapy Research*.

