# Supplementary material for: Screening of Natural Bioactive Metabolites and Investigation of Antioxidant, Antimicrobial, Antihyperglycemic, Neuropharmacological, and Cytotoxicity Potentials of Litsea polyantha Juss. Ethanolic Root Extract
Source: Evid Based Complement Alternat Med. 2017 Oct 30;2017:3701349. doi: 10.1155/2017/3701349 (PMC5682899; doi:10.1155/2017/3701349)
Supplement: Supplementary file 1 — Supplemental Table-1: DPPH Free Radical Scavenging Assay. Supplemental Table-2: Total Phenolic Content. Supplemental Table-3: Total Flavonoid content. Supplemental Table-4: Total Tannin content. Supplemental Table-5: ANOVA results of the effects of ethanol extract of roots of Litsea polyantha on glucose-loaded mice. Supplemental Table- 6: ANOVA results of neuropharmacological effect of ethanol extract of Litsea polyantha on open field test. Supplemental Table- 7: ANOVA results of neuropharmacological effect of ethanol extract of Litsea polyantha on hole Cross test. Supplemental Table-8: ANOVA results of neuropharmacological effect of ethanol extract of Litsea polyantha on hole board test. Supplemental Table-9: Diagram of zone of inhibition ± SEM of root extract against 6 bacterial strains. [file 3701349.f1.docx]

**Supplementary Material**

**Screening of Natural Bioactive Metabolites and Investigation of Antioxidant, Antimicrobial, Antihyperglycemic, Neuropharmacological and Cytotoxicity potentials of *Litsea polyantha* Juss.** **Ethanolic Root Extract**

**Nripendra Nath Biswas,^1^* Amit Kumar Acharzo,^1^ Shams Anamika,^1^ Shamsunnahar Khushi,^1^ and Bishwajit Bokshi^1^**

**^1^Pharmacy Discipline, Life Science School, Khulna University, Khulna-9208, Bangladesh.**

**Corresponding Author: Nripendra Nath Biswas; nnathbiswas@gmail.com**

**Supplemental Table-1:** DPPH Free Radical Scavenging Assay

| Concentration ( mg/mL) | Average percent inhibition of root of *Litsea polyantha* | SD | Average percent inhibition ascorbic acid | SD |
| --- | --- | --- | --- | --- |
| 0 | 1.06 | 0.304 | 6.8 | 0.141 |
| 0.301 | 1.7 | 0.601 | 15.01 | 0.304 |
| 0.602 | 5.64 | 0.601 | 21.1 | 0.141 |
| 0.903 | 11.18 | 0.757 | 56.44 | 0.148 |
| 1.204 | 25.67 | 0.148 | 65.5 | 0.608 |
| 1.505 | 38.34 | 0.453 | 74.01 | 0.304 |
| 1.806 | 50.9 | 0.177 | 90.94 | 0.304 |
| 2.107 | 59.1 | 0.304 | 96.63 | 0.742 |
| 2.408 | 67.3 | 0.332 | 97.33 | 0.75 |
| 2.709 | 71.03 | 0.304 | 98 | 0 |

Percent inhibition of DPPH Free Radical was calculated by measuring the absorbance (optical density-OD) of each concentration against a blank at 517 nm using a UV spectrophotometer and calculated using the following equation:

% inhibition of DPPH $=(1-\frac{Sample OD}{Blank OD})$ ×100

The experiment was replicated twice and standard deviation (SD) was calculated using GraphPad Prism software.

| Concentration ( mg/mL) | Average absorption | SD |
| --- | --- | --- |
| 0.2 | 1.339 | 0.001 |
| 0.15 | 0.965 | 0.006 |
| 0.1 | 0.648 | 0.006 |
| 0.08 | 0.519 | 0.003 |
| 0.06 | 0.314 | 0.001 |
| 0.04 | 0.25 | 0.003 |
| 0.02 | 0.139 | 0.008 |

**Supplemental Table-2:** Total Phenolic Content

The absorption of gallic acid at various concentrations was recorded by using the spectrophotometer at 765 nm wavelength against blank and developed a calibration curved to extrapolate the total phenolic content of the extract in mg of gallic acid equivalent (GAE) per gram of dry extract. The experiment was replicated twice and standard deviation (SD) was calculated using GraphPad Prism software.

| Average absorbance | Concentration ( mg/mL) | SD |
| --- | --- | --- |
| 0.5135 | 1 | 0.0007 |
| 0.3985 | 0.75 | 0.0004 |
| 0.244 | 0.5 | 0.0007 |
| 0.1135 | 0.25 | 0.0001 |
| 0.0125 | 0 | 0.0004 |

**Supplemental Table-3:** Total Flavonoid content

The absorption of quercetin at various concentrations was recorded by using the spectrophotometer at 510 nm wavelength against blank and developed a calibration curved to extrapolate the total flavonoid content of ethanol extract of *Litsea polyantha* root in mg of quercetin equivalent (QE) per gram of dry extract. The experiment was replicated twice and standard deviation (SD) was calculated using GraphPad Prism software.

**Supplemental Table-4:** Total Tannin content

| Concentration ( mg/mL) | Average absorbance | SD |
| --- | --- | --- |
| 0.1 | 0.1075 | 0.0007 |
| 0.2 | 0.266 | 0.0014 |
| 0.3 | 0.421 | 0.0014 |
| 0.4 | 0.5795 | 0.0007 |
| 0.5 | 0.639 | 0.0014 |

The absorption of gallic acid at various concentrations was recorded by using the spectrophotometer at 510 nm wavelength against blank and developed a calibration curved to extrapolate the total tannins of ethanol extract of *Litsea polyantha* in mg of gallic acid equivalent (GAE) per gram of dry extract. The experiment was replicated twice and standard deviation (SD) was calculated using GraphPad Prism software.

**Supplemental Table-5:** ANOVA results of the effects of ethanol extract of roots of *Litsea polyantha* on glucose-loaded mice

| Observation | F value | Degrees of freedom | P value |
| --- | --- | --- | --- |
| Blood glucose level ( mmol/L) in fasting stage | 7.60 | 3 | 0.0022 |
| Blood glucose level ( mmol/L) in 30 min | 31.61 | 3 | < 0.0001 |
| Blood glucose level ( mmol/L) in 90 min | 40.90 | 3 | < 0.0001 |
| Blood glucose level ( mmol/L) in 120 min | 57.70 | 3 | < 0.0001 |

The four groups of either sex mice (five mice/ group) were treated with vehicle (1% Tween 80 in water) at dose of 10 mL/kg , glibenclamide at the dose of 10 mg/kg, and the plant extract at 250 and 500 mg/kg body weight respectively. The blood glucose level was monitored at indicated time of first column of the table with the help of glucometer in mmol/L unit. The F value, degrees of freedom and correspondent significance level for individual experiment period were calculated by using InVivoStat Single Measure Parametric Analysis software and indicated in column two, three, and four respectively.

**Supplemental Table- 6:** ANOVA results of neuropharmacological effect of ethanol extract of *Litsea polyantha* on open field test

| Observation | F value | Degrees of freedom | P value |
| --- | --- | --- | --- |
| No. square crossed by the mice in 0 min | 1.17 | 3 | 0.3528 |
| No. square crossed by the mice in 30 min | 176.32 | 3 | < 0.0001 |
| No. square crossed by the mice in 60 min | 135.42 | 3 | < 0.0001 |
| No. square crossed by the mice in 90 min | 79.21 | 3 | < 0.0001 |
| No. square crossed by the mice in 120 min | 66.43 | 3 | < 0.0001 |
| No. square crossed by the mice in 180 min | 255.71 | 3 | < 0.0001 |

The four groups of either sex mice (five mice/ group) were given with vehicle (1% Tween 80 in water) at dose of 10 mL/kg , diazepam at the dose of 1 mg/kg, and the plant extract at 250 and 500 mg/kg body weight respectively. The locomotor activity of survival mice was monitored at indicated time of first column of the table. The F value, degrees of freedom and correspondent significance level for individual experiment period were calculated by using InVivoStat Single Measure Parametric Analysis software and indicated in column two, three, and four respectively.

**Supplemental Table- 7:** ANOVA results of neuropharmacological effect of ethanol extract of *Litsea polyantha* on hole Cross test

| Observation | F value | Degrees of freedom | P value |
| --- | --- | --- | --- |
| No. of hole crossed by the mice in 0 min | 1.12 | 3 | 0.3709 |
| No. of hole crossed by the mice in 30 min | 33.46 | 3 | < 0.0001 |
| No. of hole crossed by the mice in 60 min | 39.20 | 3 | < 0.0001 |
| No. of hole crossed by the mice in 90 min | 41.05 | 3 | < 0.0001 |
| No. of hole crossed by the mice in 120 min | 17.39 | 3 | < 0.0001 |
| No. of hole crossed by the mice in 180 min | 27.06 | 3 | < 0.0001 |

The four groups of either sex mice (five mice/ group) were given with vehicle (1% Tween 80 in water) at dose of 10 mL/kg , diazepam at the dose of 1 mg/kg, and the plant extract at 250 and 500 mg/kg body weight respectively. The locomotor activity of survival mice was monitored at indicated time of first column of the table. The F value, degrees of freedom and correspondent significance level for individual experiment period were calculated by using InVivoStat Single Measure Parametric Analysis software and indicated in column two, three, and four respectively.

**Supplemental Table-8**: ANOVA results of neuropharmacological effect of ethanol extract of *Litsea polyantha* on hole board test.

| Observation | F value | P value |
| --- | --- | --- |
| No. of head dipping by the mice in 0 min | 3.14 | 0.0544 |
| No. of head dipping by the mice in 30 min | 35.76 | < 0.0001 |
| No. of head dipping by the mice in 60 min | 44.00 | < 0.0001 |
| No. of head dipping by the mice in 90 min | 128.39 | < 0.0001 |
| No. of head dipping by the mice in 120 min | 209.33 | < 0.0001 |
| No. of head dipping by the mice in 180 min | 191.06 | < 0.0001 |

The four groups of either sex mice (five mice/ group) were given with vehicle (1% Tween 80 in water) at dose of 10 mL/kg , diazepam at the dose of 1 mg/kg, and the plant extract at 250 and 500 mg/kg body weight respectively. The exploratory behavior of survival mice was monitored at indicated time of first column of the table. The F value, degrees of freedom and correspondent significance level for individual experiment period were calculated by using InVivoStat Single Measure Parametric Analysis software and indicated in column two, three, and four respectively.

**Supplemental Table-9:** Diagram of zone of inhibition ± SEM of root extract against 6 bacterial strains

| Bacterial Strain | Zone of inhibition ±SEM for Test Extract | |
| --- | --- | --- |
|  | 250 μg/disc | 500 μg/disc |
| *Escherichia coli* | 8.75±0.25 mm | 12.25±1.25 mm |
| *Vibrio cholera* | 0 mm | 10±1 mm |
| *Salmonella typhi* | 0 mm | 8±1 mm |
| *Pseudomonas aeruginosa* | 7±1 mm | 11.25±0.75 mm |
| *Staphylococcus aureus* | 0 mm | 0 mm |
| *Staphylococcus pyogens* | 0 mm | 8.25±0.25 mm |

The antibacterial activity against aforementioned bacterial strain was determined by measuring the diameter of zone of inhibition induced by plant extract with the help of slide calipers in term of millimeter unit. Values are presented as the Mean ± SEM (n = 2).
